# Audit of methods used to contact the duty doctor - Abraham Cowley Unit

**DOI:** 10.1192/bjo.2021.916

**Published:** 2021-06-18

**Authors:** Ivan Shanley, Jessica Thomas

**Affiliations:** 1 Surrey and Borders Partnership NHS Foundation Trust; 2 Ashford and St Peter's NHS Foundation Trust

## Abstract

**Aims:**

The aim of this audit was to determine whether the duty doctor of a 4 ward inpatient psychiatric unit is contacted safely, effectively and in a manner that can be monitored. This is in line with trust protocol and the method stated is via switchboard. Should a deficit be found it was the aim to make an appropriate intervention.

**Background:**

In the Abraham Cowley Unit, there is a Senior House Officer ‘on-call’ duty doctor 24/7. The shifts are 2 x 12.5 hours daily and at all times the duty doctor should be contacted via switchboard. Contacting via switchboard is important to ensure there is an audit trail of calls made. Issues that arise from using other methods of contact, e.g. calling direct extensions, include miscommunication and the doctor not being reached in a timely manner. This had been identified as an issue anecdotally by junior doctors on call and also highlighted following an untoward incident.

**Method:**

The method by which the on call doctor was contacted was recorded in Excel for 5 consecutive 12.5 hour shifts in October 2019. The standard set for calls via switchboard was 80%. Following the initial results and the subsequent intervention, a repeat audit was performed using the same method.

**Result:**

Initial Outcome

Initially it was found that only 25% of calls received where through the appropriate channel (5 out of 20 calls). This fell far below the 80% standard and an intervention was therefore devised.

Intervention

In order to ensure that all ward staff were aware of the trust policy posters were created and placed above all ward telephones and the telephone in the assessment suite office. This information was also handed over to the nurses in charge directly in order for it to be filtered through to other staff during handover.

Post Intervention Outcome

Following the intervention 88% of calls received where through the appropriate channels (7 out of 8 calls) and the 80% standard was achieved.

**Conclusion:**

There has been a demonstrable improvement in the adherence to trust policy when contacting the duty doctor, with the percentage of calls made through the appropriate channel rising from 25% to 88%. This has now met the agreed standard of 80% and will improve the trust's ability to monitor contact of the duty doctor effectively.

